# Outcomes of heart transplantation using ECMO-supported donation in brain dead donors

**DOI:** 10.1007/s11748-025-02208-0

**Published:** 2025-10-11

**Authors:** Soojin Lee, Seunghwan Song, Hye Won Lee, Harin Rhee, Soo Yong Lee, Kyung-Hee Kim

**Affiliations:** 1 Department of Thoracic and Cardiovascular Surgery, Pusan National University School of Medicine, Biomedical Research Institute, Pusan National University Hospital, 179 Gudeok-Ro, Seo-Gu, Busan, 49241 South Korea; 2 Division of Cardiology, Department of Internal Medicine, School of Medicine, Pusan National University, Biomedical Research Institute, Pusan National University Hospital, Busan, South Korea; 3 Department of Internal Medicine, Pusan National University School of Medicine, Busan, South Korea; 4 Department of Nephrology, Biomedical Research Institute, Pusan National University Hospital, Busan, Republic of Korea; 5 Division of Cardiology, Department of Internal Medicine, and Research Institute for Convergence of Biomedical Science and Technology, Pusan National University Yangsan Hospital, Pusan National University School of Medicine, Yangsan, South Korea; 6 Division of Cardiology, Department of Internal Medicine, Incheon Sejong Hospital, Incheon, South Korea

**Keywords:** Heart transplantation, Donor, Korea, Extracorporeal membrane oxygenation, Brain death

## Abstract

**Background:**

In South Korea, extracorporeal membrane oxygenation (ECMO) is used as a bridge to optimize utilization of heart obtained from donors after brain death. However, the heart utilization rate and the effectiveness of ECMO in donation after brain death (DBD) donors, prior to donation, remain unclear. This study aimed to analyze the early postoperative outcomes of recipients who received hearts from DBD donors supported by ECMO, and to identify the factors associated with successful transplantation outcomes.

**Methods:**

Donors who received ECMO support were divided into two groups, one, whose hearts were successfully transplanted (*n* = 3), and the other, whose hearts were not utilized for transplantation (*n* = 13), at our institution between 2013 and 2024. Preoperative donor characteristics of the two donor groups were compared to identify the factors influencing successful heart transplantation. Recipients’ preoperative, intraoperative findings, and 1-year postoperative outcomes were analyzed.

**Results:**

Among 190 DBD donors, 16 (8.4%), supported by ECMO, were grouped. The transplanted heart rate in this group was 18.8% (3 out of 16 ECMO-supported potential donors). The 1-year graft survival and recipient survival rates were 100%. The transplanted donor group tended to be younger than the non-transplanted group, with a median age difference of 26 years (*p* = 0.031).

**Conclusion:**

Prior to donation, ECMO can be effectively used in brain-dead donors, to improve the rate of heart transplants. The postoperative outcomes of recipients, who received hearts procured from ECMO-supported donors, were satisfactory. Among ECMO-supported DBD donors, the median age tended to be lower in the transplanted donor group.

**Supplementary Information:**

The online version contains supplementary material available at 10.1007/s11748-025-02208-0.

## Introduction

With an increase in the requirement of heart transplants, reliance on donation after brain death (DBD) alone has proven insufficient to satisfy the demand for donor organs. In the United States (US), donation after circulatory death (DCD) was first introduced in the early 1990s. Since the 2000 s, DCD has increased globally, and is now one of the primary methods of organ donation in the US [1]. Nevertheless, according to the national data from the United Network for Organ Sharing, the median waiting time for patients with Heart Status 2 listed between 2011 and 2014 was 726 days, which was significantly longer than that in the past [2].

In South Korea, the average waiting time for heart transplantation among Status 0 patients has been increasing. In 2022, status 0 patients had to wait an average of 112 days, and in 2023, the waiting period extended by 30.4% (146 days) [3]. Considering that DCD has not yet been implemented for heart transplantation in South Korea, the shortage of donor organs is expected to present even greater challenges for transplant candidates. In this context, the most viable strategy for addressing donor shortages in Korea is to optimize the utilization of currently available donors. One approach involves the use of extracorporeal membrane oxygenation (ECMO) to maintain hemodynamic stability and organ function in donors [4].

Despite its potential, there is no standardized protocol for the application and maintenance of ECMO in brain-dead donors, and the quality of organs retrieved from ECMO-supported donors remains controversial. Consequently, the use of ECMO has not been widely adopted across all centers. Therefore, a preliminary investigation is essential to evaluate whether ECMO support can effectively enhance the survival of DBD donors.

This study aimed to analyze the characteristics of brain-dead donors who received ECMO support at our institution and to identify the factors associated with successful transplantation. In addition, we assessed the early postoperative outcomes of heart recipients from ECMO-supported donors.

## Materials and methods

### Ethical considerations

This observational clinical study did not require clinical trial registration. The research was conducted by reviewing medical records, and the analyzed data were de-identified to protect the anonymity and non-identifiability of the patients. This study was approved by the Institutional Review Boards of Pusan National University Hospital (No. 2503-011-149). The requirement for informed consent was waived because the analysis was conducted retrospectively using electronic medical records.

### Donor data collection

In accordance with national allocation policies applied across organ transplantation centers in South Korea, organs from brain-dead donors at our institution were distributed based on priority rankings, with recipients assigned to various centers, including our own. Consequently, all brain-dead donors included in this study underwent management and organ procurement at our center, whereas the procured hearts were transplanted into recipients at different transplant centers.

As no standardized criteria or methodology currently exist for the evaluation of donor cardiac function under ECMO support, preoperative assessment in this study was conducted based on a comprehensive review of daily transthoracic echocardiography findings, electrocardiogram results, and arterial waveform analyses (with the area under the waveform representing cardiac output) prior to procurement. All DBD were evaluated during ECMO support; among those supported with venoarterial (VA) ECMO, additional assessment was performed under minimal flow conditions, maintaining approximately 10% of estimated cardiac output. Medical contraindications to heart transplantation were defined as severe left ventricular dysfunction (left ventricular ejection fraction < 40%), left ventricular hypertrophy, significant regional wall motion abnormalities, and severe valvular disease (excluding cases of mild-to-moderate mitral or tricuspid insufficiency, which may be considered for pre-transplant surgical repair depending on recipient condition).

In this retrospective study, we analyzed the electronic medical records of DBD organ donors at our institution between 2013 and 2024. Among these donors, only those supported by ECMO prior to organ donation were included. Potential donors who ultimately proceeded with heart donation were identified after applying the following exclusion criteria: withdrawal of consent (*n* = 3), medical contraindications (*n* = 4), or advanced donor age (criteria vary among centers and are not absolute; at our institution, donor hearts ≤ 70 years are generally accepted, with flexibility in borderline cases) (*n* = 4). Further exclusion criteria included a poor organ status during harvesting (*n* = 1) and delayed detection of viral infections (*n* = 1). Donors whose hearts were successfully transplanted into recipients (*n* = 3) were included in the transplanted heart donor group, whereas those meeting the exclusion criteria were included in the non-transplanted heart donor group. The organ utilization and transplanted heart rates were calculated using the following formulae:

$${\text{Organ utilization rate}} = \left( {{\text{number of harvested donors}}/{\text{number of ECMO}} - {\text{supported potential donors}}} \right) \times {1}00$$

$${\text{Transplanted heart rate}} = \left( {{\text{number of transplanted donors}}/{\text{number of ECMO}} - {\text{supported potential donors}}} \right) \times {1}00.$$

For donors, data collection was comprehensive and included patient characteristics, intraoperative findings, and heart utilization details. Specifically, we recorded the demographic and clinical profiles of patients, such as, sex, age, underlying diseases, Sequential Organ Failure Assessment (SOFA) score, vasoactive–inotropic score (VIS), cardiopulmonary resuscitation (CPR) duration, cause of death determined by neurological criteria, ECMO details, serum lactate levels, heart utilization rates, heart status via visual inspection, and duration of hospital and intensive care unit (ICU) stay.

### Recipient data collection

The data were collected from electronic medical records, encompassing both inpatient and outpatient records, for a minimum period of 1 year postoperatively. Data collected included patient characteristics, intraoperative details, and postoperative outcomes. The recorded variables included demographics, clinical profiles (age, body mass index, smoking history, underlying diseases, preoperative left ventricular ejection fraction, prior cardiac surgeries, pre-transplant status, diagnosis, and preoperative ECMO use), and ischemic time of the transplanted heart. Intraoperative details included operative, cardiopulmonary bypass (CPB), and aortic cross-clamp (ACC) times. Postoperative outcomes assessed were in-hospital mortality, 1-year mortality, postoperative complications, echocardiographic findings, lengths of hospital and ICU stay, ventilator days, and readmission rates.

### Comparison between transplanted and non-transplanted donors

In this study, ECMO-supported donors were categorized into two groups, based on whether the heart was transplanted into the recipient or not. The transplanted donor group comprised donors whose hearts were successfully utilized for transplantation. The non-transplanted donor group included those whose hearts were not utilized, as defined by the exclusion criteria outlined above. A comprehensive comparative analysis was conducted between the two groups, focusing on preoperative patient characteristics.

### Statistical analysis

The baseline characteristics of the two donor groups were compared, and recipients’ preoperative, intraoperative data, and postoperative outcomes were assessed. For continuous variables, comparisons were made using either the independent *t* test or the Wilcoxon rank-sum test. Categorical variables were analyzed using the Chi-square or Fisher’s exact test. Statistical significance was set at a *p* value of less than 0.05. All statistical analyses were performed using R, version 4.2.2 (R Core Team, 2020).

## Results

### Utilization of hearts from ECMO-supported DBD donors

**Fig. 1:**
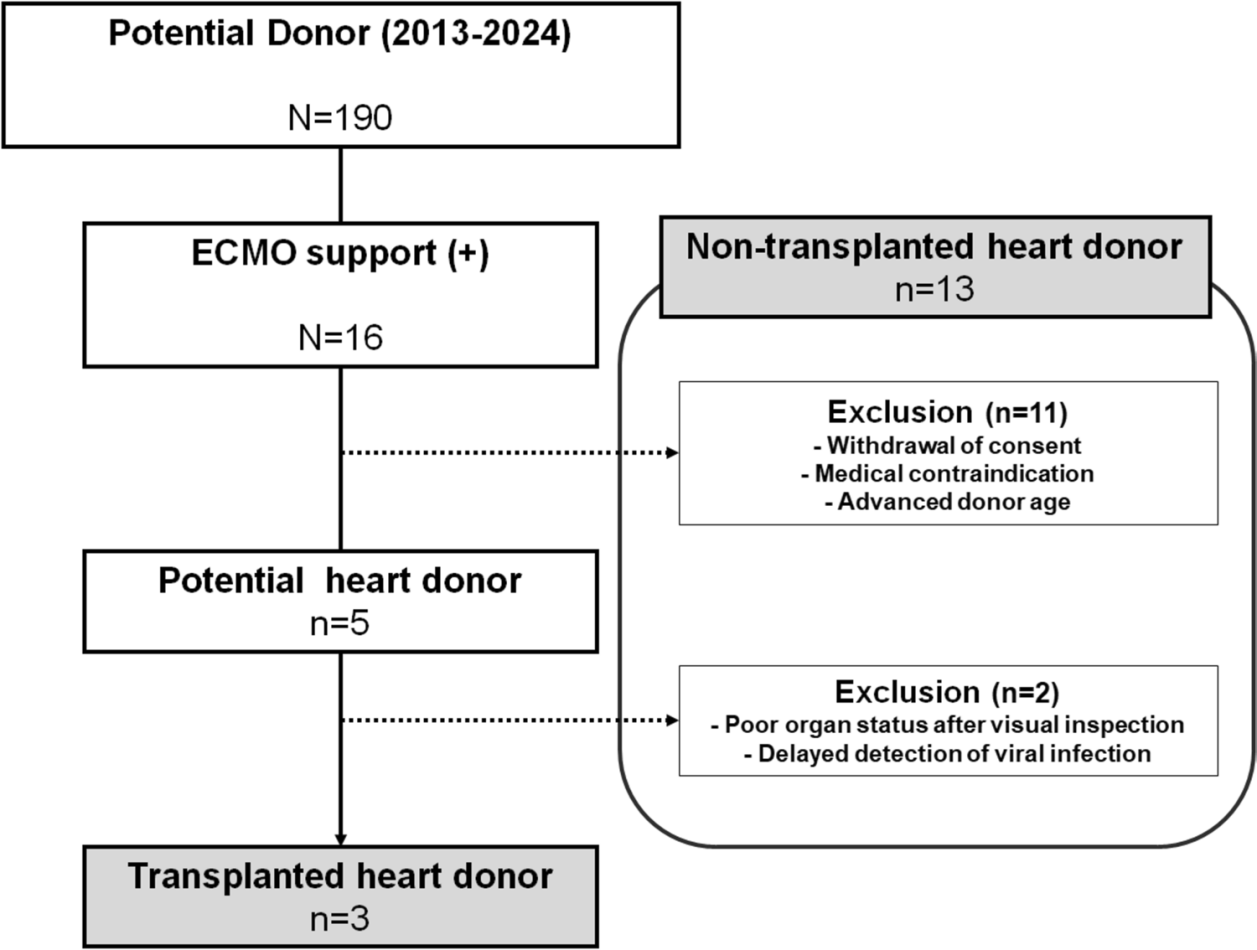
Flowchart of the study. Among 190 brain-dead potential donors registered at our center between 2013 and 2024, 16 received ECMO support before heart retrieval. Of these, 11 were excluded due to withdrawal of consent by family, medical contraindications, or advanced donor age judged unacceptable by recipient centers. The remaining 5 were considered potential heart donors. Among these, 2 donor hearts were not transplanted because of poor gross appearance at retrieval or unexpected positive viral markers. Finally, 3 donor hearts were successfully transplanted (transplanted heart donors). Donors whose hearts were not ultimately used for transplantation were categorized as non-transplanted heart donors (*n* = 13). *ECMO* extracorporeal membrane oxygenation

Over the 12-year period, 16 brain-dead donors at our institution received ECMO support as a potential DBD heart donors, representing an ECMO application rate of 8.4% (*n* = 16). Among these, five patients were identified as potential donors, all of whom underwent heart harvesting, resulting in an organ utilization rate of 31.3%. However, one heart was deemed unsuitable during intraoperative visual inspection owing to poor conditions, and another was excluded because of a late-detected viral infection confirmed by blood tests at the time of retrieval (Fig. 1). Consequently, hearts from three donors were successfully transplanted, yielding an organ transplantation rate of 18.8% (Table 1).

**Table 1:** Early outcomes of heart utilization from donors following extracorporeal membrane oxygenation

		***N* = 16**
Potential donors		5
Harvested donors		5
Transplanted donors		3
Cause of donation failure	Poor organ status after visual inspection	1
	Delayed detection of viral infection	1
Organ utilization rate, %		31.3
Transplanted organ rate, %		18.8
Recipient’s survival, 1 year		100% (*n* = 3/3)
Graft survival, 1 year		100% (*n* = 3/3)

The organ utilization rate was calculated as the proportion of actual organ use among all donors. The transplanted organ rate was determined by the proportion of actual organs used among potential donors. Values are presented as numbers (%)

### Comparison of baseline characteristics between transplanted and non-transplanted donor groups

**Fig. 2:**
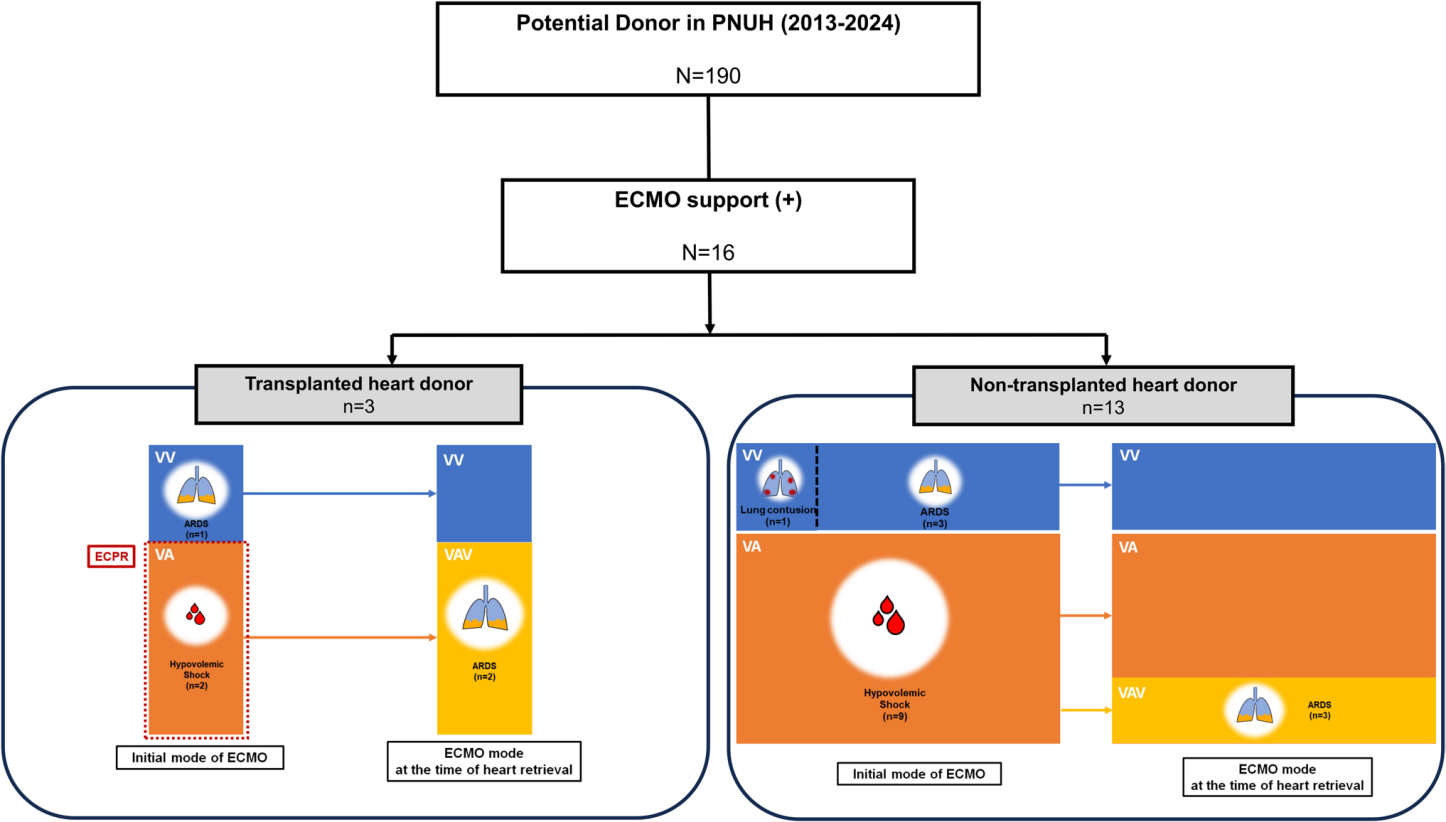
Indications for ECMO support and ECMO mode conversion prior to heart donation in brain-dead donors. ECMO indications, initial ECMO modes, and modes at the time of heart retrieval in ECMO-supported brain-dead donors between 2013 and 2024 (*n* = 16). Donors were categorized into transplanted heart donor group (*n* = 3) and non-transplanted heart donor group (*n* = 13). In the transplanted heart donor group, one donor with ARDS initially received VV ECMO, and two donors with hypovolemic shock received VA ECMO during ECPR. All two donors with initially supported with VA ECMO were converted to VAV ECMO before heart retrieval due to progressive ARDS. In the non-transplanted donor group, VV ECMO was applied for lung contusion or ARDS (*n* = 4), while VA ECMO was applied for hypovolemic shock (*n* = 9). Among the latter, three donors required conversion to VAV ECMO before heart retrieval because of ARDS progression. *ECMO* extracorporeal membrane oxygenation, *ECPR* extracorporeal cardiopulmonary resuscitation, *VV* venovenous, *VA* venoarterial, *VAV* venoarterial venous, *ARDS* acute respiratory distress syndrome

A total of 16 ECMO-supported DBD heart donors were divided into transplanted (*n* = 3) and non-transplanted (*n* = 13) donor groups. In both groups, the majority of patients received ECMO support following the diagnosis of brain death. Only two patients, both in the transplanted donor group, initially received VA ECMO as part of extracorporeal CPR at presentation and were subsequently declared brain dead. In both groups, VA ECMO was initiated due to hypovolemic shock resulting from multiple trauma. All of these patients subsequently underwent a mode conversion to veno-arterial-venous (VAV) ECMO during their hospital course (Fig. 2). The transplanted donor group appeared younger than the non-transplanted group (24 [13–34] years vs. 50 [11–76] years, *p* = 0.031). The non-transplanted donor group showed a higher proportion of male donors, a greater prevalence of underlying diseases, and a higher incidence of brain death due to trauma (Table 2).

**Table 2:** Comparison of baseline characteristics between successful and unsuccessful heart transplantations after ECMO support

Variables			Total (*N* = 16)	Transplanted donor (*n* = 3)	Non-transplanted donor (*n* = 13)	Effect size (95% CI)	*p* value	Test statistic
Age, years			47 [11–76]	24 [13–34]	50 [11–76]	MD + 23.5 (+ 6.2 to + 40.8); Cohen’s d = 1.07	0.031	2.66
Sex, male			12 (75%)	2 (66.7)	10 (76.9)	OR 0.60 (0.04–8.99)	0.788	17.5
Underlying disease	Hypertension		2 (12.5%)	0 (0)	2 (15.4)	OR 0.92 (0.03–26.2)	0.558	22.5
	Diabetes mellitus		2 (12.5%)	0 (0)	2 (15.4)	OR 0.92 (0.03–26.2)		
	Other		1 (6.3%)	0 (0)	1 (7.7)	OR 2.00 (0.05–74.9)		
SOFA score			12.4 ± 2.9	13 ± 3.6	12.3 ± 2.7	MD –0.69 (–6.08 to + 4.69); Cohen’s d = –0.23	0.821	−0.252
Vasoactive–inotropic score			122 [0–2784]	122 [0–572]	122 [0–2784]	MD + 268.7 (–276.2 to + 813.5); Cohen’s d = + 0.37	0.839	21.5
Transfer from an outside hospital for organ donation			5 (31.3%)	1 (33.3)	4 (33.8)	OR 1.13 (0.08–16.3)	1.000	19
Diagnosis of death by neurologic criteria, d			3.4 ± 2.2	3.7 ± 1.2	3.4 ± 2.4	MD −0.28 (–2.13 to + 1.56); Cohen’s d = –0.12	0.585	15
Cause of death by neurologic criteria	Trauma		12 (75%)	2 (66.7)	10 (77)	OR 0.60 (0.04–9.16)	0.585	15.5
	Hypoxic brain damage		2 (12.5%)	1 (33.3)	1 (7.7)	OR 6.00 (0.26–140.0)		
	Cerebrovascular accident		1 (6.3%)	0 (0)	1 (7.7)	OR 2.00 (0.05–74.9)		
	Other		1 (6.3%)	0 (0)	1 (7.7)	OR 2.00 (0.05–74.9)		
CPR before donation			11 (68.8%)	2 (66.7)	9 (69.2)	OR 0.89 (0.06–12.9)	0.948	–0.071
ECPR			2 (12.5%)	2 (66.7)	0 (0)	OR 52.0 (1.28–2117.7)	1.000	-
CPR duration, min			10 [0–86]	36 [5–67]	10 [0–86]	MD –7.46 (–51.5 to + 36.6); Cohen’s d = –0.29	-	-
ECMO details	Support duration, d		2.1 ± 0.5	1.7 ± 0.6	2.2 ± 0.4	MD + 0.50 (–0.21 to + 1.21); Cohen’s d = 1.15	0.144	20
	Peripheral configuration		16 (100%)	3 (100%)	13 (100%)	-	0.749	21
	Initial mode	VA	11 (68.6%)	2 (66.7)	9 (69.2)	OR 0.89 (0.06–12.9)	1.00	20
		VV	5 (31.3%)	1 (33.3)	4 (30.8)	-		
	Mode change		5 (31.3%)	2 (66.7)	3 (23.1)	OR 6.67 (0.44–101.7)	0.181	11
	Usage of anticoagulation		8 (50%)	2 (66.7)	6 (46.2)	OR 2.33 (0.17–32.6)	0.587	15.5
Hospital length of stay, d			5.3 ± 3.1	4.0 ± 1.0	5.5 ± 3.3	MD + 1.54 (–0.62 to + 3.70); Cohen’s d = + 0.49	0.891	21
ICU length of stay, d			5.3 ± 3.1	4.0 ± 1.0	5.5 ± 3.3	MD + 1.54 (–0.62 to + 3.70); Cohen’s d = + 0.49	0.891	21
Lactate, mmol/L	Before ECMO support		8.4 [1.4–15]	11.9 [3–15]	7.2 [1.4–15]	MD –1.43 (–9.35 to + 6.50); Cohen’s d = –0.24	0.746	−0.353
	After ECMO support		6.5 [1.1–14]	6.5 [4.2–15]	5.5 [1.1–14]	MD –1.16 (–8.94 to + 6.61); Cohen’s d = –0.17	0.612	11.5

Values are presented as means ± standard deviation, medians [range], or numbers (%)

*SOFA* Sequential Organ Failure Assessment, *CPR* cardiopulmonary resuscitation, *ECMO* extracorporeal membrane oxygenation, *VA* venoarterial, VV venovenous, *DNC* death by neurologic criteria, *CI* confidence interval, *MD* mean difference, *OR* odd ratio

### Preoperative and intraoperative details of heart transplant recipients

The three heart transplant recipients (recipients 1, 2, and 3) who received hearts from donors with ECMO support were aged between 56 and 69 years. Except for recipient 2, the other two patients had at least one pre-existing comorbidity. All three recipients received mechanical circulatory support as a bridge to transplantation. Recipient 1 was supported with peripheral VA ECMO, whereas the other two were on left ventricular assist devices, corresponding to pre-transplant statuses of 0 and 1, respectively.

The average cold ischemic time was 147 min, all shorter than 4 h. The mean CPB time was 145 min and the average total operative time was 335 min. However, ACC times were unavailable for two recipients because of incomplete records (Table 3).

**Table 3:** Preoperative and intraoperative details of the heart transplant recipients

			Recipient 1	Recipient 2	Recipient 3
Preoperative					
	Age, years		56	64	69
	BMI, kg/m^2^		23.24	24.96	20.94
	Smoking history		+	−	−
	Underlying disease				
		Hypertension	+	−	-
		DM	+	−	−
		Others	−	−	+, CKD
	Preoperative LVEF, %		23	20	28
	Previous cardiac surgery		-	LVAD	LVAD
	Pretransplant status		0	1	1
	Diagnosis		ICMP	DCMP	ICMP
	ECMO application		+	−	−
	Cold ischemic time, min		91	156	194
Intraoperative					
	CPB, min		158	99	178
	ACC, min		87	?	?
	Operative time, min		300	282	424

*ECMO* extracorporeal membrane oxygenation, *BMI*, body mass index, *DM* diabetes mellitus, *LVEF* left ventricular ejection fraction, *CPB* cardiopulmonary bypass, *ACC* aortic cross-clamping, *ICMP* ischemic cardiomyopathy, *DCMP* dilated cardiomyopathy, *LVAD* left ventricular assist device, *CKD* chronic kidney disease

### Postoperative outcomes of heart transplant recipients

None of the three heart transplant recipients experienced in-hospital or 1-year mortality. There were neither any major adverse cardiovascular or cerebrovascular events (MACCEs), nor hospital readmissions, 1 year postoperatively. However, recipient 3, who had pre-existing chronic kidney disease (CKD), developed acute kidney injury (AKI) during the immediate postoperative period, necessitating short-term continuous renal replacement therapy (CRRT). The patient subsequently recovered and was discharged without requiring long-term dialysis.

The length of ICU stay for all the three patients was less than 1 week, with a mean total hospital stay of 42 days. During their ICU stay, mechanical ventilation was used for an average of 8.7 h. On the 1-year transthoracic echocardiogram follow-up post-transplantation, all the three recipients demonstrated normal left ventricular contractility (Table 4).

**Table 4:** Postoperative outcomes of heart transplant recipients

		Recipient 1	Recipient 2	Recipient 3
Hospital stay, days		71	24	31
ICU stay, days		6	3.5	3
Ventilator, hr		11.2	4.4	10.6
Complication		−	−	AKI
In−hospital mortality		−	−	−
1−year mortality		−	−	−
1−year MACCEs		−	−	−
Readmission		−	−	−
Postoperative TTE	LVEF, %	65	65	65
	RWMA	−	−	−

*ECMO* extracorporeal membrane oxygenation, *ICU* intensive care unit, *MACCE* major adverse cardiac and cerebrovascular events, *TTE* transthoracic echocardiography, *LVEF* left ventricular ejection fraction, *RWMA* regional wall motion abnormality

## Discussion

Approximately 20% of the organs from DBD donors are discarded because of hemodynamic instability before organ procurement [5, 6]. ECMO has the potential to enable organ donation in patients who might otherwise be at high risk of death. This study demonstrates that ECMO support effectively increases the utilization rate of hearts from DBD donors. Among the brain-dead donors managed at our institution, 8.4% of potential heart donors required pre-donation ECMO support. Remarkably, 18.8% of these cases resulted in successful heart transplantation, underscoring the value of ECMO in optimizing donor heart utilization.

Donor shortage remains a critical issue worldwide. Although the number of heart transplants performed annually is steadily increasing, the number of patients with advanced heart failure is also growing at an even faster pace, leading to prolonged waiting times for transplantation. Increasing the utilization rate of available donors could mitigate the impact of donor shortages; however, current donor utilization rates remain suboptimal. Globally, donor utilization rates have been reported to be below 50% [7–9]. In South Korea, although utilization rates are gradually improving, they were reported to be only 42.9% in 2019 [10]**.** Therefore, two primary strategies exist to address donor shortages: expanding the donor pool and increasing the utilization rate of existing donors. Regarding the former, the concept of DCD has been introduced in several countries [11–13]. However, legal, ethical, and systemic barriers have hindered their adoption in South Korea. Given these constraints, providing pre-donation ECMO support is currently the most viable approach for preserving donor organ utilization in South Korea. Our results highlight the critical role of ECMO in maximizing the effectiveness of a limited donor pool, providing a tangible strategy to address the pressing issue of donor shortage.

ECMO provides temporary circulatory support to stabilize donor hemodynamics. However, concerns persist regarding the functional viability of hearts retrieved from ECMO-supported donors. Peripheral VA ECMO may result in left ventricular unloading and can lead to Harlequin syndrome, which causes upper body ischemia, including coronary ischemia [14]. In addition, VA ECMO is associated with a systemic inflammatory response that may contribute to end-organ dysfunction, including myocardial injury [15], as well as ischemia–reperfusion injury [16]. Previous retrospective analyses have reported a 25% discard rate of kidneys from ECMO-supported donors, with 11.8% discarded without clear medical contraindications, indicating a potential bias [17]. Therefore, we sought to analyze the outcomes of organs retrieved from ECMO-supported donors and identify the factors influencing transplantation success, aiming to address these concerns and enhance donor organ utilization.

The mode change rate was 31%, suggesting that proactive adjustments were made to optimize donor management and improve organ utilization rates. 66.7% of patients in the transplanted group initially received VA ECMO but underwent a mode change to VAV ECMO due to hypoxia detected on arterial blood gas analysis from the right radial artery, indicative of poor lung function. This adjustment successfully mitigated coronary hypoxia, preserved cardiac function, and enabled heart transplantation. Therefore, rather than excluding heart utilization solely based on the application of VA ECMO, our findings suggest a promising approach: in donors who initially required VA ECMO due to transient hemodynamic instability, timely mode conversion and thorough cardiac function assessment following short-term ECMO support may contribute to improving heart utilization rates. After a short duration of ECMO, the hearts were harvested, and it was observed that successfully transplanted donor group’s age was relatively younger. Old age is an independent risk factor for non-utilization in transplantation, and the findings of this study exhibit a similar trend [10]. However, the sample size is too small to determine whether younger age is an independent factor associated with successful transplantation. Therefore, a comprehensive assessment is essential to maximize organ utilization and to avoid the premature exclusion of potentially transplantable hearts from ECMO-supported donors.

Successful early postoperative outcomes were confirmed in heart transplant recipients who received hearts from ECMO-supported donors. The 1-year survival rate was 100%, with no reported cases of MACCEs. Among the three patients, one developed AKI requiring CRRT, which was attributed to pre-existing CKD. The outcomes of this study were comparable with the most recent 1-year survival rate of 84.5% reported in the ISHLTR [18]. Given the limited data available on the comprehensive 1-year MACCE rate in heart transplant beneficiaries and considering that long-term outcomes are significantly influenced by factors such as chronic allograft vasculopathy, malignancy, infection, acute rejection, and renal insufficiency, further long-term analyses are warranted to understand the fate of ECMO-supported hearts and their recipients [19].

Heart transplant outcomes are influenced not only by the functional status of the donor but also by the characteristics of the recipient. Compared with previous studies on large cohorts of heart transplant recipients, the recipients included in this study were older on average and had longer CPB times. Despite these restraints, early postoperative outcomes in the recipients were comparable to those typically observed in heart transplant patients [13]. This favorable outcome may be attributed to the relatively short cold ischemic times and the use of appropriate ECMO support before organ retrieval, which helped maintain donor hemodynamic stability. However, to accurately understand the interplay between donor and recipient factors and their impact on transplant outcomes, further studies with larger sample sizes that comprehensively analyze both donor and recipient variables are necessary.

This study has some limitations. This was a single-center, retrospective study with a very limited number of cases and insufficient data on both donors and recipients. As such, the results should be interpreted as descriptive and hypothesis-generating, and confirmation through larger multi-center analyses will be essential. In addition, the current Center for the Korean Network for Organ Sharing registry contains only basic information about donors and lacks detailed classification and outcome data for recipients who received transplants from these donors. Therefore, a more detailed and integrated data entry system must be established to enable future studies based on a robust national database. Despite these limitations, our study benefitted from the active collaboration of the recipient hospital, allowing us to access early postoperative outcome data for recipients. This collaboration enabled a preliminary analysis of the outcomes and provided valuable insights. Furthermore, a key strength of this study is its focus on ECMO-supported donors. As few studies exist on this topic, our detailed and comprehensive analysis of ECMO-supported DBD donors and transplant outcomes significantly contributes to the understanding of the utilization of ECMO in donors.

In summary, ECMO can be an effective method for increasing heart utilization rates in heart transplantation. The recipients of hearts retrieved from ECMO-supported donors demonstrated favorable early postoperative mortality and morbidity. Furthermore, the transplanted group showed a trend toward younger donor age. Given the small sample size, this observation needs to be explored in larger studies. A detailed national database is crucial to evaluate the role of ECMO in heart transplantation.

## Supplementary Information

Below is the link to the electronic supplementary material.

Supplementary file1 Comparison of transplanted organ rate between ECMO-supported and Non-ECMO-supported brain-dead donors. *ECMO* Extracorporeal membrane oxygenation (TIF 269 KB)

## Data Availability

The datasets generated and analyzed during the current study are not publicly available due to privacy of patient data but are available from the corresponding author on reasonable request and after approval by the Institutional Review Board of the hospital (No. 2503-011-149).

## Abbreviations

ECMO: Extracorporeal membrane oxygenation
DBD: Donation after brain death
DCD: Donation after circulatory death
VA: Venoarterial
SOFA: Sequential Organ Failure Assessment
VIS: Vasoactive-inotropic score
CPR: Cardiopulmonary resuscitation
ICU: Intensive care unit
CPB: Cardiopulmonary bypass
ACC: Aortic cross-clamping
VAV: Veno-arterial-venous
MACCEs: Major adverse cardiovascular or cerebrovascular events
CKD: Chronic kidney disease
AKI: Acute kidney injury
CRRT: Continuous renal replacement therapy
ISHLTR: International Society of Heart and Lung Transplantation Registry
