# Supplementary figures and images for: Outcomes of heart transplantation using ECMO-supported donation in brain dead donors
Source: Gen Thorac Cardiovasc Surg. 2025 Oct 11;74(3):262–70. doi: 10.1007/s11748-025-02208-0 (PMC12957031; doi:10.1007/s11748-025-02208-0)

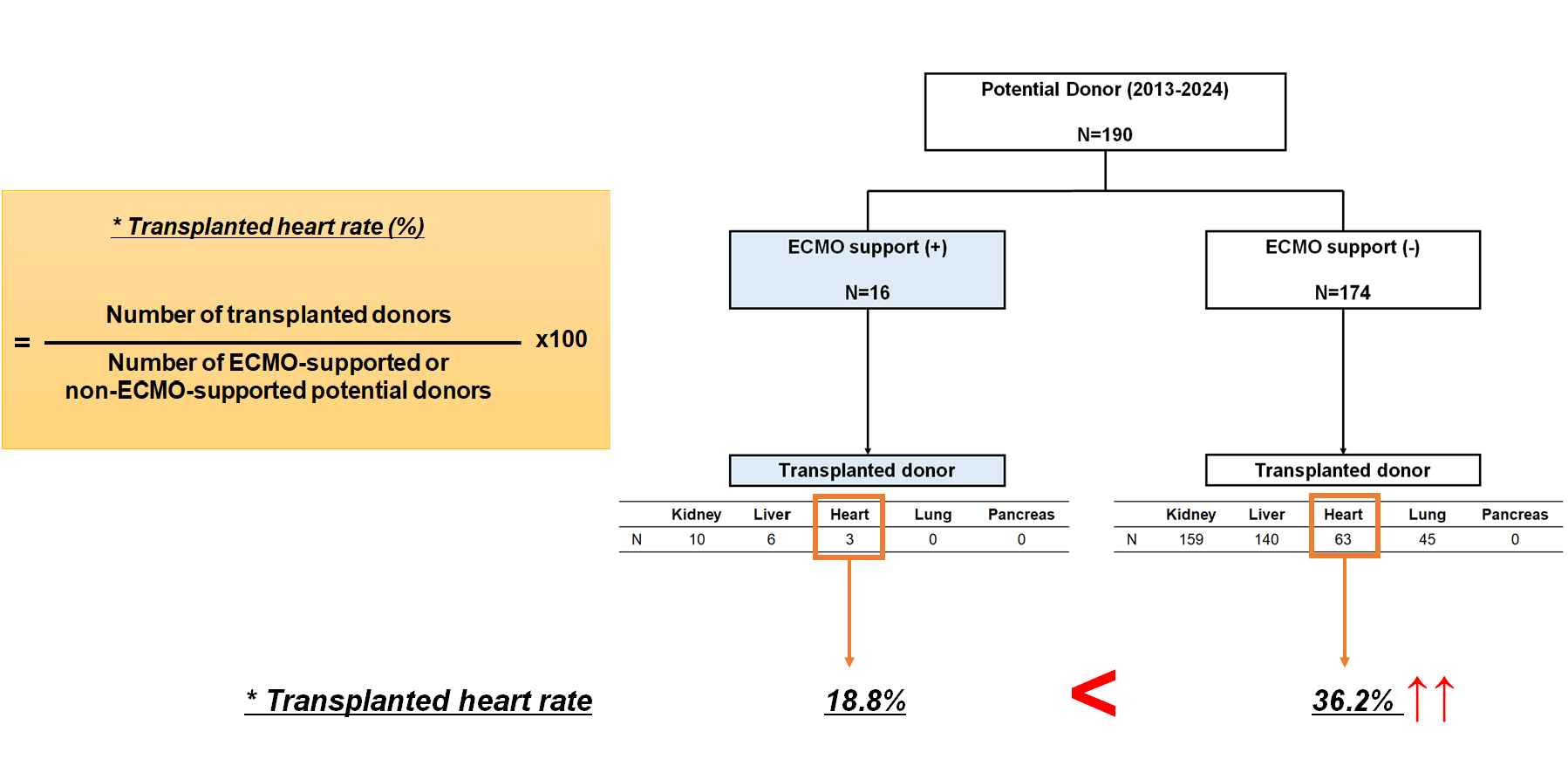

Supplement: Supplementary file 1 — Supplementary file1 Comparison of transplanted organ rate between ECMO-supported and Non-ECMO-supported brain-dead donors. ECMO Extracorporeal membrane oxygenation (TIF 269 KB) [file 11748_2025_2208_MOESM1_ESM.tif]
